# Linking diabetic retinal changes with the occurrence of anxiety and depression in working population

**DOI:** 10.1515/med-2026-1377

**Published:** 2026-03-06

**Authors:** Sonja P. Cekić, Dijana S. Risimić, Maja M. Simonović, Nikola M. Stojanović, Aleksandra Ignjatović

**Affiliations:** Department of Ophthalmology, Faculty of Medicine, University of Niš, Niš, Serbia; Clinics for Ophthalmology, University Clinical Centre Niš, Niš, Serbia; Department of Ophthalmology, Faculty of Medicine, University of Belgrade, Belgrade, Serbia; Department of Psychiatry, Faculty of Medicine, University of Niš, Niš, Serbia; Center for Mental Health Protection, University Clinical Centre Niš, Niš, Serbia; Department of Statistics, Faculty of Medicine, University of Niš, Niš, Serbia

**Keywords:** diabetes, macula, retina, anxiety, depression

## Abstract

**Objectives:**

Diabetic retinopathy (DR) is a severe complication of diabetes mellitus (DM) leading to vision loss and blindness, which typically develops 12–20 years after the diagnosis of DM. Anxiety and depression frequently co-occur with chronic medical conditions like DM and DR, impacting their onset, course, and prognosis. This study aimed to examine the link between anxiety, depression, DR, and macular changes.

**Methods:**

A cross-sectional study was conducted at the Clinic for Ophthalmology, University Clinical Center Niš, Serbia, involving 80 adult participants, naïve to psychiatric and invasive ophthalmological therapy, divided into two groups: 40 patients with DR, and 40 healthy controls. Ophthalmic examinations, biochemical measurements, and assessments of anxiety and depression using the Hospital Anxiety and Depression Scale (HADS) and Montgomery and Asberg Depression Rating Scale (MADRS) were performed.

**Results:**

Results indicated that patients with DR had significantly higher levels of anxiety and depression compared to controls. The study found no significant correlation between HADS and MADRS scores with patients’ gender, type of therapy, or the type of DR. However, a higher HADS and MADRS score was observed in patients with left-side OCT macular edema.

**Conclusions:**

The study highlights the complex interaction between psychiatric disorders and somatic illnesses, emphasizing the need for comprehensive care strategies that address both physical and mental health to improve patient outcomes.

## Introduction

Diabetic retinopathy (DR) is a severe microvascular complication of diabetes mellitus (DM) that can lead to vision loss and blindness. It is a leading cause of irreversible blindness and visual impairment among working-age individuals worldwide [1]. Microvascular complications in retinal tissue typically develop 12–20 years after the diagnosis of type 2 diabetes mellitus (DMT2) [2], 3]. In the early nonproliferative stages (NPDR), visual symptoms are often mild and may be easily overlooked. As the disease progresses to severe nonproliferative or proliferative diabetic retinopathy (PDR), symptoms become more pronounced. Diabetic macular changes, which can occur at any stage of the disease, affect central visual acuity [2]. These macular changes can lead to significant loss of central vision and result in a high level of disability in daily functioning. Even though the disease is partially understood, there are limited diagnostic and therapeutic options for DR, and some risky and not completely safe treatment options include injection of anti-VEGF drugs [2].

Anxiety can be defined as a future-oriented fear characterized by a complex system of cognitive, emotional, physiological, and behavioral responses, all of which are geared towards preparing for anticipated events or situations that are perceived as threatening [4]. Depression is a mental health disorder affecting emotional, cognitive, and physical well-being which is characterized by persistent feelings of sadness, hopelessness, and a lack of interest or pleasure in activities that were once enjoyable [5]. Anxiety and depression frequently go hand in hand with chronic medical conditions, such as DM and its complications. These psychiatric disorders may have an impact on the onset, course, prognosis, and treatment of DMT2. The interaction of mood disorders and DM and DR is bidirectional and results in different levels of disability, deterioration of quality of life, and premature death. Anxiety and depression in diabetic patients who are suffering from DMT1 or DMT2 have been evaluated in different studies, and a close relationship has been confirmed [6], 7]. A recent study provides support that DM is associated with the likelihood of having anxiety disorders and elevated anxiety symptoms [8]. A combined study reported that patients with DR are more prone to developing anxiety and depression than those with DM without complications [9].

Despite having a close relationship between mood disorders and poor glycemic control, the pathophysiological link between mood disorders and DR and macular changes in DM remains still unclear. Thus, our study aimed to examine the link between the occurrence and extent of anxiety and depression in patients with DR and macular changes and to compare the obtained results with the ones from the control group subjects.

## Materials and methods

### Study design and data collection

The study is designed as a cross-sectional study and conducted at the Clinic for Ophthalmology, University Clinical Center Niš, Serbia. The study design was supported by previous publications with similar construct [10]. It included 80 adult working-aged individuals, divided into two groups. The first group, 40 patients with the diagnosis of DMT2 and proven DR, and the second comprised 40 healthy subjects who reported for a routine eye examinations and those coming for free ophthalmological examinations organized by our institution. The presence of DM was confirmed based on medical records, increased fasting glycemia, or the use of antidiabetic therapy (oral hypoglycemic drugs and/or insulin). None of the patients was submitted to any kind of interventions for their DR prior to this study.

The exclusion criteria were intraocular inflammatory diseases (scleritis, uveitis), inherited macular disorders, retinal vascular anomalies and vascular occlusive diseases, and glaucoma and age-related macular degeneration, smoking, use of angiotensin receptor blockers, antioxidants or mineral supplements, and any previous ophthalmic surgical or laser interventions.

### Assessment of diabetic retinopathy and diabetic macular changes

In all subjects, the ophthalmic examination attained the following: best corrected visual acuity (BCVA) based on the Early Treatment of Diabetic Retinopathy Study (ETDRS), applanation tonometry, anterior segment and posterior segment examination by indirect ophthalmoscopy, fundus photography.

Photofundus check-up,color and green mode, as well as fluorescein angiography (Visu Cam Lite Digital Camera) were performed in all patients with DR, having their pupils dilated; under the same conditions, by the same digital fundus camera and by the same ophthalmologist. Optical coherent tomography of the macula and optical coherence angiography (RTVue XR Avanti, AngioVue, Optovue, Inc., Freemont, CA) conducted by the same ophthalmologist, as well.

ETDRS classification was used for the staging of the DR and diabetic macular edema in the study group without changes, and the study group with mild (background) and advanced diabetic retinopathy [2]. The presence of diabetic macular edema (DME) was noted as none, central-involved or noncentral-involved DME. Central foveal thickness (CFT) was measured on the right and left eye.

The Optovue AngioVue System (ReVue XR software version 2017.1.0.151, issued by Optovue Inc., Fremont, CA, USA) was used to obtain optical coherence tomography angiography (OCTA) images after conducting the standardized protocol whose basis is the SSADA (split-spectrum amplitude decorrelation algorithm) as described previously [11]. The classification of DR according to the ETDRS was used as guidance for macular capillary network visualization, with scans performed centered on the fovea area (3 × 3 mm) over the macular region divided into fovea, parafovea, and the full image. The automatic vascular network segmentation of the deep capillary plexus (DCP), superficial capillary plexus (SCP), and choriocapillaris was performed with the application of the AngioAnalyticTM software.

The 3D Projection Artifact Removal (PAR) algorithm [12] was used to improve OCTA image quality. Images with residual motion artifacts, low centration and focus, incorrect segmentation, and the index of the signal strength below 40 were excluded from the analysis. The same software calculated vessel density (VD), which is represented as the percentage of the microvasculature-occupied area and thickness in the total area of the scan and all of the sections. The presence of vascular dropout in the SCP and DSP was noted.

### Serum biochemical analysis

Fasting blood samples were taken to measure blood glucose, glycosylated hemoglobin A_1c_ (HbA_1c_), total cholesterol, HDL, LDL, and triglycerides using an automated biochemical analyzer (Olympus AU680, Beckman Coulter, Los Angeles, USA).

### Assessment of anxiety and depression

After the ophthalmological examination patients were examined by psychiatrist expert (M.S.) who conducted a psychiatric interview and estimated patients’ mental status according to the guidelines of the international disease classification 10 (ICD-10). Depression and anxiety were assessed by the Hospital Anxiety and Depression Scale (HADS) and Montgomery and Asberg Depression Rating Scale (MADRS). The severity of depressive episode in the studded patients’ population was done using MADRS as was the case in previous studies [13] conducted by the clinical.

The HADS Scale included 14 items assessing anxiety and 7 items assessing depression. Each item has a four-point Likert scale, ranging from 0 (no, definitely not) to 3 (yes, definitely). Six items are scored reversely, as suggested by the instructions. The total score is the sum of each item’s scores with higher scores reflecting more severe depression symptoms. HADS has been proven to be an effective tool for the assessment of anxiety and depression either in or developing countries with good reliability and validity [14]. The inter-rater reliability was calculated using the Intraclass Correlation Coefficient (ICC), which yielded a high ICC value of 0.91.

The MADRS is the clinician-rated 10-item scale with the following items: 1) apparent sadness; 2) reported sadness; 3) inner tension; 4) reduced sleep; 5) reduced appetite; 6) concentration difficulties; 7) lassitude; 8) inability to feel; 9) pessimistic thoughts; and 10) suicidal thoughts. Answers to all items are given on the 7-point Likert scale ranging from 0 (not at all) to 6 (definitively), with higher scores reflecting more severe depression symptoms. The total score is the sum of all answered items [14]. The calculated ICC was found to be 0.95.

### Statistical analysis

Data are presented as mean and standard deviation or in the form of absolute and relative numbers. The Shapiro-Wilk test was used to evaluate data distribution. Comparison of continuous variables between two groups was performed by Students’ t-test or Mann-Whitney test, depending on data distribution. The comparison of continuous variables between the groups was performed by the Kruslal-Walli’s test. The association of HADS and MADRS scores with demographic and clinical characteristics was examined by correlation (Spearman’s rank correlation coefficient) and regression analysis (multivariate linear regression). Comparison of categorical features was performed using Chi-square and Fisher’s test with a significance threshold set at p<0.05. Statistical data processing was done in the R and RStudio software packages.

### Ethics statement

All participants in the study gave written informed written consent at the beginning of the study during the examination at the ophthalmologist. The study was performed in accordance with the rules and was approved by the Internal Ethics Committee of the Faculty of Medicine in Niš and of Clinical Centre Niš (8-19-01-007/13-012 from November 2013).

## Results

A total of 80 subjects equally distributed in two groups, with no age and sex difference amongst them, were included in this study (Table 1). The groups of subjects with DR had diabetes for an average of 17.25 years. Around 58 % of subjects with DR were on insulin therapy, 25 % used oral antidiabetics, and around 18 % were on combined oral and insulin therapy. In the group of subjects with DR significant increase in blood glucose, glycosylated HbA_1c_, as well as lipid parameters, cholesterol, HDL, and LDL concentration, was noted (Table 1). Also, values of the systolic and diastolic blood pressure were found to be significantly higher in the group of patients with DR (Table 1).

**Table 1:** Demographic and clinical characteristics in two study groups.

Parameter/group	DR (n=40)	Control (n=40)	p^a^
Age	54.5 ± 8.2	55.5 ± 6.3	>0.05

Gender			

Male	27 (67.5 %)	20 (50 %)	>0.052^b^
Female	13 (32.5 %)	20 (50 %)	

Biochemical parameters			

Blood glucose	8.8 ± 2.6	4.7 ± 0.7	<0.001
HbA_1C_	8.2 ± 1.7	4.9 ± 0.7	<0.001
HDL	1.7 ± 0.3	1.6 ± 0.3	0.017
LDL	3.9 ± 0.4	3.2 ± 0.6	<0.001
Cholesterol	5.8 ± 1.2	4.03 ± 0.7	<0.001
Triglycerides	1.3 ± 0.6	1.3 ± 0.5	>0.05

Systolic blood pressure	138.3 ± 13.4	125.6 ± 10.4	<0.001
Diastolic blood pressure	86.5 ± 11.1	77.3 ± 10.2	<0.001

DR, diabetic retinopathy; ^a^Students t-test, ^b^Chi-square test.

The values of BCVA on the right and left eye, as well as CFT right and left, were found to be statistically significantly lower in patients with DR compared to the control group (p<0.001, Table 2). Also, OCT macular edema on the left and right occurred statistically significantly more often in affected patients than in the control group (p<0.001). The central-involved macular edema predominated on the left side (52.5 %), and the noncentral type was the most common on the right side (47.5 %) in subjects belonging to the DR group. The dropout occurred only in DR subjects, and the superficial dropout was most common on the left eye (87.5 %), followed by deep dropout on the right (67.5 %) and left eye (65.0 %) (Table 2). The type of DR was statistically significantly different compared to the control group (p<0.001).

**Table 2:** Clinical parameters in the study and control group.

Parameter/Group	DR (n=40)	Control (n=40)	p^a^
Visual acuity			

BCVA on right eye	0.71 ± 0.44	1 ± 0	<0.001
BCVA on the left eye	0.61 ± 0.48	1 ± 0	<0.001

CFT			

Right	348.7 ± 82.4	246.7 ± 5.7	<0.0012^b^
Left	362 ± 85.5	248.1 ± 6.5	<0.0012^b^

OCT macular edema right			

None	5 (12.5 %)	40 (100 %)	<0.0013^c^
Center involved	16 (40 %)	0	
Noncenter involved	19 (47.5 %)	0	

OCT macular edema left			

None	3 (7.5 %)	40 (100 %)	<0.0013^c^
Center involved	21 (52.5 %)	0	
Noncentral involved	16 (40 %)	0	

OCTA dropout right			

Superficial	23 (57.5 %)	0	<0.0014^d^
Deep	27 (67.5 %)	0	<0.0014^d^

OCTA dropout left			

Superficial	35 (87.5 %)	0	<0.0014^d^
Deep	26 (65 %)	0	<0.0014^d^

Type of DR			

No changes	0	40 (100 %)	<0.0014^d^
NPDR mild	11 (27.5 %)	0	
NPDR advanced	29 (72.5 %)	0	

^a^Mann-Whitney test, ^b^Students t-test, ^c^Chi-square test, ^d^Fishers test; CFT, central foveal thickness.

Levels of anxiety and depression estimated using HADS and MADRS scores were found to be statistically significantly higher in patients with DR compared to the control group (p<0.001) (Table 3).

**Table 3:** The degree of depression and anxiety in the study and control group.

Parameter/Group	DR (n=40)	Control (n=40)	p^a^
HADS	18.3 ± 8	12.1 ± 3.2	<0.001
MADRS	16.2 ± 9.8	5.8 ± 1.7	<0.001

^a^Mann-Whitney test; HADS, hospital anxiety and depression scale; MADRS, montgomery and asberg depression rating scale.

It was observed that HADS and MADRS scores did not show statistically significant differences based on patients’ gender, type of therapy, or type of DR or in relation to dropout of the right eye (p>0.05, Table 4). However, HADS and MADRS scores were found to be statistically significantly higher in patients with deep OCTA dropout and OCT edema, on the left side, respectively compared to those without changes (Table 4).

**Table 4:** Association between HADS and MARDS scores and features of DR.

Parameter/group	HADS	MARDS
Gender		

Male	17.7 ± 7.9	15.1 ± 9.3
Female	19.4 ± 8.5	18.7 ± 10.5
p-Value^a^	>0.05	>0.05

Therapy		

Oral	18.5 ± 7.8	18 ± 7.8
Insulin	16.9 ± 7.9	13.8 ± 9.5
Combined	22.6 ± 8.1	21.7 ± 11.4
p-Value^b^	>0.05	>0.05

Type of DR		

NPDR mild	17.6 ± 7.4	14.5 ± 10.5
NPDR advanced	18.5 ± 8.3	16.9 ± 9.6
p-Value^a^	>0.05	>0.05

OCTA dropout right		

Superficial		

Yes	18.9 ± 7.8	17.9 ± 8.7
No	17.5 ± 8.5	13.9 ± 10.9
p-Value^a^	>0.05	>0.05

Deep		

Yes	18.7 ± 8.1	17.3 ± 9.3
No	17.4 ± 9.3	14 ± 10.7
p-Value^a^	>0.05	>0.05

OCTA dropout left		

Superficial		

Yes	18.9 ± 8.3	17.2 ± 9.8
No	13.4 ± 1.9	9.6 ± 7.3
p-Value^a^	>0.05	>0.05

Deep		

Yes	20.1 ± 8.2	15.9 ± 9.6
No	14.7 ± 6.8	16.3 ± 10.2
p-Value^a^	0.042	>0.05

OCT macular edema right		

Yes	19.09 ± 8.24	16.60 ± 9.58
No	12.60 ± 2.41	13.60 ± 11.72
p-Value^a^	>0.05	>0.05

OCT macular edema left		

Yes	18.8 ± 8.2	17 ± 9.7
No	12.3 ± 2.1	6.3 ± 3.2
p-Value^a^	>0.05	0.040

^a^Mann-Whitney test, ^b^Kruskal-Walli’s test.

In the correlation analysis, it was determined that HADS and MADRS are not statistically significantly related to age (p=0.743, respectively p=0.858), blood glucose levels (p=0.365, respectively p=0.133), and HbA_1c_ (p=0.587, that is, p=0.612). The multivariate model showed that the values of the MADRS score are influenced by BVCA (p=0.040) corrected for other parameters in the model (Table 5).

**Table 5:** Association of HADS and MADRS with demographic and clinical parameters – multiple linear regression analysis.

Parameter	B	SE	Beta	p-Value
HADS				

Age	−0.077	0.159	−0.078	0.633
Blood glucose	−0.500	0.658	−0.163	0.452
HbA_1c_	0.530	1.066	0.110	0.622
BCVA on the left eye	−4.435	2.860	−0.268	0.130
OCT macular edema left	0.840	2.121	0.064	0.695

MADRS				

Age	0.015	0.177	0.012	0.934
Blood glucose	−0.524	0.734	−0.140	0.481
HbA_1c_	−1.054	1.188	−0.180	0.381
BCVA on the right eye	−6.895	3.237	−0.343	0.040
OCT macular edema left	9.675	5.625	0.265	0.095

B, unstandardized regression coefficient; SE, standard error; Beta, standardized regression coefficient.

## Discussion

Diabetes is associated with a range of complications due to chronic high blood sugar levels. These complications can include cardiovascular diseases, neuropathy, nephropathy, retinopathy, foot problems, skin conditions, hearing impairment, mental health issues, etc. Proper management of diabetes through medication, lifestyle changes, and regular monitoring can help mitigate these complications [15]. Sometimes these complications could mutually overlap and exacerbate/cause the other. The occurrence of complications in younger patients with DM, below the age of 65, as the ones included in this study (Table 1), must be prevented since this would greatly improve their life quality. Interestingly, although DR and other complications of DM are associated with older age [15] in the present study the levels of depression and anxiety were not found to correlate significantly with age (Table 5).

Associations between blood lipids in patients with DR and chest pain have been previously described in the literature, validating the association between abnormal lipid metabolism and fundus microcirculation alterations [16]. The mentioned dyslipidemia is associated with changes in blood vessels nourishing the heart muscle. In the same light, one might expect that in patients with DR with affected lipid metabolism (Table 1), atherosclerotic changes in the blood vessels of the brain might reflect brain functioning. Brain changes in metabolic disorders such as DM include glucose and lipid metabolism abnormalities, oxidative stress, mitochondrial dysfunction, and protein changes, which are the bottom line associated with Alzheimer’s disease through the promotion of amyloid-beta pathology [17]. As one of the complications of DM is the changes in brain white and gray mass structure, the results of a European American cohort study from 2016 involving 655 patients indicated that anxiety and depression might be following these structural changes [18]. Thus, patients with altered lipid metabolism and DM and suffering from a DR as a complication might be predisposed to anxiety and depression (seen through an increase in HARD and MARDS, Table 3). Interestingly, in a multiple linear regression analysis HARD and MARDS did not correlate with lipid changes nor with the changes in blood pressure (Table 5). These observations could potentially be viewed as a consequence of a small sample size, but also due to the rigorous inclusion and exclusion criteria applied to the experimental group.

There are reports on the occurrence of anxiety and depression in patients with DR more than in other patients with DM [9]. However, reports also suggest that anxiety and depression often co-occur [19]. These patients tend to experience more severe symptoms, poorer outcomes, and greater use of healthcare resources compared to those with only one disorder [20]. Individuals who are depressed and anxious are less likely to adhere to the additional demands of diabetes self-care recommendations. They tend to be less physically active, are less likely to follow their dietary regimen, and are less likely to take prescribed medications, causing complications of DM [21]. Also, the impairment in the function of the CNS could be associated with the impairment in energy generation and spending, as has been suggested [22].

The interaction between psychiatric disorders and somatic illnesses and their complications is complex and bidirectional, and the comorbidity between DM and depression has not fully been identified. There has been an increased risk of depression in people with DM and an increased risk of DM in patients with depressive disorder [23]. Recent studies have also associated sibling recurrence risk and familial clustering of type 2 diabetes with inflammatory genes, which may contribute to the development of depressive disorders [24]. The relationship between depression and the complications of DM, such as the one studied here, is unlikely to be explained by a straightforward stress-disease model, and it is more plausible that shared risk factors act through various biological pathways. It is known that DM affects the hypothalamic-pituitary-adrenal axis causing symptoms of depression [25], but also dysfunction in the endocrine system could cause changes in the retina [26]. The connection between the retina and the hypothalamus via the hypothalamoretinal tract, has revealed that this brain structure has an immense impact on the eye, affecting different structures within the eye. Also, the hormones secreted by the hypophysis, adrenocorticotropic hormone (ACTH), have a potential impact on DR [26]. Only a tiny portion of this connection might be observed through the correlation between MDARS and a decrease in BCVA on the left eye (Table 5). Furthermore, one of the items on MARDS evaluate sleep quality, which was found to generally more altered in patients with DM, suggesting potential impairment of retinal signaling in melatonin secretion [27].

One of the important findings of this study is the significant correlation between MARDS with unilateral (left) macular edema on the left eye (Table 4). The changes of on the left eye and increase in MARDS scores appear paradoxes to some extent, but not unreported [28]. The asymmetric visual impairment can point to the more pronounced disability perceived by the patient, than in the case of a symmetrical impairment, causing greater distress and generalized sense of helplessness. One can potentially connect the observed left eye asymmetry (macular or nasal area damage) with the imbalance in the right cortical areas, through a diminished input from the left eye and increased vulnerability of right cortical areas altering negative emotion perception and expression (e.g. sadness) and integration of visuospatial and emotional context [29]. Thus, speculating we can suggest that changes in the left macular area functioning directly impacts right cortex, which reflect a depressive state (higher MARDS) in the examined patient population.

According to previous experience with visual loss or the present pressing visual loss makes, patients with DR prone to the development of anxiety. The levels of anxiety in patients with DR were found to be significantly higher than in the control group subjects (Table 3). Although the HADS scores in patients with DR were high, indicating increased anxiety in these patients, the HADS scores were also slightly higher than normal values, which are expected to be up to 7 according to cut off values [30]. These data indicate that anxiety is increased in subjects referring to an ophthalmologist for an eye examination, potentially due to eminent examination of the eyesight. Also, it is interesting to mention that patients with choroidal changes, seen as deep dropout on the left eye, have been found to have statistically significantly higher HADS scores (Table 4).

Previous studies showed that there is an association between DM and mild to moderate anxiety levels [8], 21], 31]. In a study conducted on a Chinese population suffering from DR, it was found that around 14 % had increased levels of anxiety, which was associated with social support, stress, a family history of DM, and some other chronic conditions [32]. Interestingly, although HADS was significantly increased in patients with DR there was no association between HADS with any of the parameters attributed to the complications of retina seen in patients with DM (Table 5). However, even though no associations were found, that does not reduce the need for interventions, support, and stress reduction in patients suffering from DR, which eventually leads to blindness. Potentially longitudinal studies examining the impact of different psychotherapeutic interventions (predominantly supportive and focused on emotional regulation) on diseases progression and overall functionality and life quality of patients with DR would contribute to better understanding between the mental state and DR. As said, these studies would not only focus on resolving current mental issues, such as depression and anxiety, but would also help patients mentally adapt their somatic state associated with both DM and DR.

Chronic hyperglycemia in DR triggers excessive oxidative stress and inflammatory cytokine release (IL-1β, IL-6, TNF-α), leading to neurovascular unit damage and degeneration [33], 34]. These processes impair neurovascular coupling by reducing nitric oxide bioavailability and disrupting glial-vascular signaling, resulting in inadequate metabolism, ischemia, and retinal neurodegeneration [35]. Similar oxidative-inflammatory and neurovascular abnormalities occur in limbic and prefrontal brain regions involved in emotional regulation, contributing to altered synaptic plasticity, HPA-axis dysregulation, and neurotransmitter imbalance associated with anxiety and depression [36], 37] These data suggesting that the changes in mentioned regions might be also seen in patients with DR contributing to the development of anxiety and depression symptoms. Epidemiological and imaging studies show that microvascular changes in DR correlate with cerebral microvascular dysfunction and a higher prevalence of affective disorders, suggesting that DR reflects a systemic neurovascular vulnerability relevant to anxiety and depressive pathology [38].

### Strengths and limitations of the study

The present study has several limitations. The first limitation of this study was the small sample size. However, the post hoc power analysis indicated that the study achieved a power of 99 % based on the comparison of HADS and MADRS values between groups, suggesting a clinically relevant effect size with the variation associated with the dataset. The second limitation is the lack of a multicentre approach which partially limits more general conclusion, while at the same time the single-centre design allowed for greater control over data collection. One should bear in mind that the patients studied are ophthalmology therapy naïve, meaning that they have never been treated with any method which could compromise eye tissue. Also, patients are naïve to psychiatric treatment, which all together decreases the potential interference with the obtained results. One might also say that the strength of this study is the population itself, since it involves relatively young people, still working, which should be a target for research and prevention of DR and its consequences on mental health.

## Conclusions

In conclusion, based on the results from a study conducted in the University Clinical Centre Niš, the relationship between diabetes and its complications, including diabetic retinopathy, anxiety, and depression, are found to be complex and multifaceted, involving various biological pathways and shared risk factors. One of the interesting and unexpected finding is the correlation between depression and left eye macular changes. The comorbidity of diabetes and psychiatric disorders exacerbates disease management challenges, leading to poorer outcomes and increased healthcare utilization. Therefore, comprehensive care strategies addressing both physical and mental health aspects are essential for improving patient outcomes in those with diabetes and its complications. Future studies should focus on designing and implementing strategies for the management of mental health issues arising from diabetes and its complication, but also on designing other psychotherapeutic approaches that would be beneficial for patient’s daily functionality and life quality.
